# Association of parent-child relationship quality and problematic mobile phone use with non-suicidal self-injury among adolescents

**DOI:** 10.1186/s12888-023-04786-w

**Published:** 2023-05-01

**Authors:** Huiqiong Xu, Wan Xiao, Yang Xie, Shaojun Xu, Yuhui Wan, Fangbiao Tao

**Affiliations:** 1 Department of Maternal, Child and Adolescent Health, School of Public Health, Anhui Medical University, No 81 Meishan Road, Hefei, 230032 Anhui China; 2 Key Laboratory of Population Health Across Life Cycle (Anhui Medical University), Ministry of Education of the People’s Republic of China, No 81 Meishan Road, Hefei, 230032 Anhui China; 3 NHC Key Laboratory of Study on Abnormal Gametes and Reproductive Tract, No 81 Meishan Road, Hefei, 230032 Anhui China; 4 Anhui Provincial Key Laboratory of Population Health and Aristogenics, 81 Meishan Road, Hefei, 230032 Anhui China

**Keywords:** Parent-child relationship, Problematic mobile phone use, Non-suicidal self-injury, Adolescents, Sex

## Abstract

**Background:**

Non-suicidal self-injury behavior (NSSI) is a common mental health threat among adolescents. Poor parent-child relationship (PCR) and problematic mobile phone use (PMPU) are risk factors for NSSI. We aimed to explore the impact of PCR quality, PMPU, and their interaction effects on NSSI among adolescents in China, as well as the sex difference.

**Method:**

A survey was conducted among school students in 4 provinces in China between 2017 and 2018. The study included 14,500 valid participants. The students’ general demographic characteristics was collected, and further data on PCR quality, PMPU, and NSSI were obtained through self-rated questionnaire. Chi-square test, binomial logistic regression models, and the Andersson Excel were used for data analysis.

**Results:**

The 12-month prevalence of NSSI was 27.3%. Lower PCR quality and PMPU were significantly associated with NSSI, respectively. The low PCR + yes PMPU group had the greatest association with NSSI, followed by the high PCR + yes PMPU group, low PCR + no PMPU group. Moreover, in low father-child relationship + yes PMPU group, females had a higher risk of NSSI than males; in high mother-child relationship + yes PMPU group, females had a higher risk of NSSI than males. Additive interaction analysis indicated that mother-child relationship quality and PMPU were associated with increased risks of NSSI, in the subgroup of males.

**Conclusions:**

The findings underline the importance of simultaneously studying the quality of PCR and PMPU for a comprehensive understanding of NSSI behavior, and especially highlights the significance of maternal relationship quality.

**Supplementary Information:**

The online version contains supplementary material available at 10.1186/s12888-023-04786-w.

## Introduction

Non-suicidal self-injury behavior (NSSI) refers to the intentional self-directed destruction of body tissue without suicidal intention and is not socially or culturally sanctioned [1]. It has been prevalent among children and adolescents worldwide, with an aggregate lifetime and 12-month prevalence of 22.1% (95% CI: 16.9–28.4%) and 19.5% (95% CI: 13.3–27.6%), respectively, between 1989 and 2018 [2]. Prior literature collected 11,814 children aged 9 to 10 years from the United States, the approximate rates were 9.1% (95% CI, 8.1-10.3%) for NSSI [3]. Moreover, a nationwide survey of 14,820 Chinese students aged 10–20 years showed 26.1% reported NSSI in the last year [4]. These behaviors were considered typical of adolescents, a manifestation of youthful resistance, and a short-lived behavior, many parents do not know the best way to approach NSSI in their children [5]. Furthermore, current research indicated that the effects of NSSI may continue into adulthood, increasing the risk of suicidal thoughts and behaviors [6]. Thus, NSSI are major public health problems in adolescents worldwide, and it was classified as a prelude to suicide or a type of personality disorder [7].

The parent-child relationship (PCR) is a relatively stable relationship model formed by the interaction between children and their parents, which has a significant impact on the children’s development. The biosocial model pointed that early vulnerability and family environment risk factors may give rise to more extreme emotional and behavioral dysregulation, such as NSSI [8, 9]. Numerous studies have indicated that functioning of the family and PCR quality were related to NSSI and psychological behavior in adolescents [10, 11]. Besides, research has demonstrated that the attachment qualities with parents in young adolescents without NSSI experiences are characterized by more trust, communication, and closeness. Conversely, poor attachment relationships with parents were associated with engaging in NSSI or they maintain it [12]. Furthermore, there were significant differences in the frequency and severity of self-injurious behavior at different levels of parent-child attachment [13].

In addition, the heterogeneity across studies on mother-child relationship (MCR) and father-child relationship (FCR) has not been unified [14, 15]. One study showed that no systematic differences in the way child-mother and child-father attachment predicted the development of externalized behavior in boys and girls [15]. Attachment theory indicates both adolescents’ attachment relationships with their mother and their father can be different and complementary, mothers are often viewed as safe haven attachment figures, whereas fathers are considered as facilitators of children’s exploration system [16]. A recent study found that the FCR and MCR have different mediating mechanisms in triggering self-injury behavior, the former relies on emotional coping style, while the latter is associated with emotional experiences, which is in line with the two main theories used to explain self-injury behavior are the interpersonal or systematic models, as well as the emotion regulation model [17]. Combine with Chinese cultural background, there may be differences in the ways in which mother and father interact with their children, fathers may be disengaged from their children’s activities, while mothers may be more involved in their children’s lives [18]. At present, this conclusion are not conclusive, for this reason, looking at relationship with mother and father separately makes sense.

In present times, the use of mobile phones has become a common phenomenon worldwide. According to studies on children and adolescents aged 9–16 years in the United States and European countries, 46–85% of them owned a mobile phone [19] Related domestic research showed that the rate of adolescents’ dependence on mobile phone use was 22.9–25.4% [20, 21]. Despite abundant benefits of this new technology, there are many concerns about its potential disadvantages. A serious concern among all is the problematic mobile phone use (PMPU), which may be one of the potential contributing factors for mental and behavioral problems in adolescents [22, 23]. Many researchers reported that PMPU was associated with NSSI and suicidal behavior [20, 23]. Thus, poor quality PCR and PMPU are both risk factors for NSSI.

Many previous studies suggested that a variety of psychological and behavioral problems in adolescents often do not exist alone, but appear to be clustered and interrelated [20, 24]. Not surprisingly, there also be high correlated between PCR quality and PMPU, PCR quality could negatively predict smartphone use disorder or internet addiction among adolescents [25, 26]. The PCR had effects on PMPU through loneliness, escape motivation and relationship motivation [27]. Based on uses and gratifications theory, social media such as mobile phones were often used to satisfy one’s unmet needs [28]. Adolescents with negative PCR tend to overuse internet to seek emotional warmth and support, thus satisfying their unmet needs in the family [29]. Longitudinal study results showed that the improvement of PCR and life satisfaction can reduce adolescents’ mobile phone addiction [30]. So it is necessary to further explore the interaction effects of PCR quality and PMPU on NSSI in adolescents.

Finally, despite evidence differentiates between males and females in terms of the prevalence and effects of PMPU [21], the perceived PCR quality [31], and the presentation of NSSI [20]. Whereas, up to now, no studies have been undertaken to examine sex differences in the interaction between PCR and PMPU on NSSI. Therefore, the present study proposes three hypotheses and validates: (1) low FCR/MCR and PMPU would be associated with a higher risk of engaging in NSSI, (2) the combined/interaction effect between low FCR/MCR and PMPU increase the risk of NSSI, and (3) the associations are stronger in females than in males.

## Methods

### Participants and procedure

A cross-sectional survey was conducted among middle school students in grades 7–12, from November 2017 to January 2018. The participants were selected from four provinces in China, namely Shenzhen City in Guangdong Province, Zhengzhou City in Henan Province, Nanchang City in Jiangxi Province, and Guiyang City in Guizhou Province. These provinces broadly represent China’s average population in terms of economic development, demographic distribution, and cooperation with our adolescent health research team. In each region, we randomly selected 4 urban and 4 rural middle schools. At least 400 students were surveyed in each school, making the sample size to be 3,200 per region. The next step was to sample the grade and class, three classes were selected from each grade for the survey, if the three classes were insufficient for the sample size, they are randomly sampled in the adjacent class until the sample size is sufficient. Subsequently, 15,486 students were asked to fill out an anonymous questionnaire. After screening, 14,500 valid samples were ultimately obtained, with 645 students refusing to participate, 226 students being absent, and 115 incomplete questionnaires, which included missing values > 5%, logic errors or participants over the age of 20, and an efficiency rate of 93.6%. More detailed information on this study has been reported previously [32].

## Measures

### Sociodemographic profile

Data on participants’ demographic information, sex (male or female), boarding school or not, grade (middle or high school), only child or not, rural or urban residency, self-assessment of the family economy, educational level of the parents, and the number of close friends were collected.

### Parent-child relationship (PCR)

PCR was measured using FACES III (Family Adaption and Cohesion Evaluation Scales III) subscales [33]. The Chinese version of the scale was translated and revised by Zhang et al. [34], which consists of two parts: FCR and MCR. Each section has 10 items, with a self-rated 5-point Likert scale ranging from 1 (little) to 5 (almost always). Such as “My father [mother] and I supported each other during difficult times”, “My father [mother] and I feel very close to each other,” and “My father [mother] and I avoid each other at home” (reversed). A higher score on the scale indicates a better level of PCR. The 75th percentile was used as the cut-off point. In the paternal relationship scale, those with scores ≥40 were defined as the higher quality group. In the maternal relationship scale, those with scores ≥49 were defined as the higher quality group. In the present study, Cronbach’s alpha for paternal relationship and maternal relationship scales were 0.851 and 0.900, respectively.

### Problematic mobile phone use (PMPU)

PMPU was evaluated using the Self-rating Questionnaire for Adolescent Problematic Mobile Phone Use [35, 36]. The scale consists of 13 items divided into three dimensions: withdrawal symptoms, craving, and physical and mental health status. Each item is rated on a 5-point Likert scale (1 = never to 5 = always), with total scores ranging from 13 to 65. The 75th percentile was the entry point, and those with scores ≥ 23 were defined as having PMPU. The Cronbach’s alpha coefficient for this questionnaire was 0.910.

### Non-suicidal self-injury (NSSI)

NSSI was measured using the Adolescent Non-Suicidal Self-Injury Assessment Questionnaire [37]. Participants were asked, “Did you have the following behaviors that caused you to intentionally hurt yourself in the past year? This behavior is not for suicide, but may cause bleeding, bruising, or pain.” A list of 12 NSSI methods were specified: pinched or scratched yourself, banged your head or fist against something, hit yourself, prick or stab yourself, cut yourself, bitten yourself, pulled your own hair, burned yourself, rub skin to bleed or bruise and engrave words or symbols on the skin. The answers to all options listed under this question were of the type “yes” or “no.” If the participants answered “yes” (one or more times), they were judged to have NSSI behavior. The Cronbach’s alpha coefficient for this questionnaire was 0.919.

### Statistical analysis

In this study, we used EpiData 3.1 to establish a database and SPSS 23.0 software package for statistical analysis. Measurement data are expressed as the mean ± standard deviation. Chi-square tests were used to compare the incidence of NSSI among sociodemographic characteristics. Binomial logistic regression models were used to examine the associations of NSSI with PCR and PMPU individually, and then in combination. In multi-factor logistic regression model, covariates were sex, boarding school, single child, grade, residency, family economy, parents’ education level, and number of friends. Furthermore, the sex differences in the associations were examined via two odds ratio (RORs, ratio of two odds ratios) [38]. Finally, the Excel additive interaction calculation table established by Thomas Anderson was used to calculate the relative excess risk of interaction (*RERI*), attributable proportions (*AP*), and synergy index (*SI*) [39]. *P* < 0.05 were considered indicative of statistically significant findings in two-sided tests.

## Results

### Characteristics of participants

In the survey of 14,500 participants, 7347 (50.7%) were male and 7153 (49.3%) were female, aged between 10 and 20 years (mean ± SD: 14.83 ± 1.79 years), with more than 98.7% of participants aged 12–18 years. Overall, 3964 (27.3%) adolescents reported NSSI in the past year; males had significantly greater tendency for NSSI behaviors than females (28.4% vs. 26.2%, *P* = 0.002), and middle school students were more prone to NSSI behaviors than high school students (30.4% vs. 24.3%, *P* < 0.001). Other sociodemographic characteristics were shown in Table 1.

**Table 1:** The frequency characteristics of NSSI in different socio-demographic variables among Chinese adolescent, n (%)

Characteristics	N = 14,500	NSSI		*χ* ^*2*^	*P*
		No(n = 10,536)	Yes(n = 3964)		
Sex				9.22	0.002
Male	7347(50.7)	5257(71.6)	2090(28.4)		
Female	7153(49.3)	5279(73.8)	1874(26.2)		
Grade				68.33	< 0.001
Middle school	7247(50.0)	5044(69.6)	2203(30.4)		
High school	7253(50.0)	5492(75.7)	1761(24.3)		
Boarding school				21.84	< 0.001
Yes	6830(47.1)	5088(74.5)	1742(25.5)		
No	7670(52.9)	5448(71.0)	2222(29.0)		
Single child status				1.99	0.158
Only child	4669(32.2)	3428(73.4)	1241(26.6)		
Non-only child	9831(67.8)	7108(72.3)	2723(27.7)		
Residency				0.001	0.974
Rural	6881(47.5)	4999(72.6)	1882(27.4)		
Urban	7619(52.5)	5537(72.7)	2082(27.3)		
Family economic				38.02	< 0.001
Poor	2039(14.1)	1375(67.4)	664(32.6)		
Fair	10,010(69.0)	7405(74.0)	2605(26.0)		
Good	2451(16.9)	1756(71.6)	695(28.4)		
Father’s education level				17.98	< 0.001
Primary school	2195(15.1)	1538(70.1)	657(29.9)		
Middle school	4706(32.5)	3370(71.6)	1336(28.4)		
High school	4120(28.4)	3064(74.4)	1056(25.6)		
College or above	3479(24.0)	2564(73.7)	915(26.3)		
Mother’s education level				15.25	0.002
Primary school	3315(22.9)	2351(70.9)	964(29.1)		
Middle school	4664(32.2)	3355(71.9)	1309(28.1)		
High school	3786(26.1)	2832(74.8)	954(25.2)		
College or above	2735(18.9)	1998(73.1)	737(26.9)		
Number of friends				84.87	< 0.001
None	437(3.0)	262(60.0)	175(40.0)		
1 ~ 2	3099(21.4)	2128(68.7)	971(31.3)		
3 ~ 5	6151(42.4)	4498(73.1)	1653(26.9)		
≥ 6	4813(33.2)	3648(75.8)	1165(24.2)		

Note: NSSI = non-suicidal self-injury.

The most frequently reported forms of NSSI were, in descending order, fist against something (20.8%), pinched yourself (15.0%), pulled your own hair (10.0%), banged your head (9.9%), scratched yourself (9.8%), engrave words or symbols on the skin (9.4%), hit yourself (8.0%), bitten yourself (7.7%), prick or stab yourself (5.4%), cut yourself (5.1%), rub skin to bleed or bruise (3.9%), burned yourself (1.4%). (Table S1).

### Independent effects of PCR and PMPU on NSSI, and sex difference

Table 2 showed that higher rates of NSSI were observed in lower FCR and MCR quality groups (*χ*^*2*^ *=* 126.36, *P* < 0.001; *χ*^*2*^ *=* 62.86, *P* < 0.001) and those with PMPU (*χ*^*2*^ *=* 272.19, *P* < 0.001). After controlling for confounding variables, multi-factor logistic regression analysis showed that FCR quality (OR = 1.54, 95% CI: 1.40–1.69), MCR quality (OR = 1.45, 95% CI: 1.33–1.58), and PMPU (OR = 1.89, 95% CI: 1.74–2.05) were also associated with NSSI (Table 2). Besides, no sex difference were found in the independent effects of PCR on NSSI, with then exception of the PMPU having a stronger effect in females than in males (Table 3).

**Table 2:** Number, percent and odds ratio of NSSI by level of parent-child relationship and PMPU in the total sample

Variables	N(%)			Model 1		Model 2		Model 3
	NSSI < 1	NSSI ≥ 1		*OR*(95%*CI*)^a^		*OR*(95%*CI*)^b^		*OR*(95%*CI*)^c^
FCR								
High	2917(79.8)	737(20.2)		1.0		1.00		1.00
Low	7619(70.2)	3227(29.8)		1.68(1.53–1.84)^*^		1.61(1.46–1.76)^*^		1.54(1.40–1.69)^*^
MCR								
High	3867(76.7)	1176(23.3)		1.00		1.00		1.00
Low	6669(70.5)	2788(29.5)		1.38(1.27–1.49)^*^		1.40(1.29–1.52)^*^		1.45(1.33–1.58)^*^
PMPU								
No	8067(76.4)	2493(23.6)		1.00		1.00		1.00
Yes	2469(62.7)	1471(37.3)		1.93(1.78–2.09)^*^		2.00(1.85–2.17)^*^		1.89(1.74–2.05)^*^

Note: FCR = father-child relationship; MCR = mother-child relationship; PMPU = problematic mobile phone use; NSSI = non-suicidal self-injury. ^a^ Unadjusted model; ^b^ Adjusted for sex, boarding school, single child, grade, residency, family economic, parents’ education level, number of friends. ^c^ Adjusted for sex, boarding school, single child, grade, residency, family economic, parents’ education level, number of friends, FCR, MCR and PMPU. ^*^P < 0.001.

**Table 3:** Number, percent and odds ratio of NSSI by level of parent-child relationship and PMPU in females and males, and the sex ratio

Variables	Females			Males			Ratio of two odds ratios in girls versus boys
	n(%)	*OR*(95%*CI*)^c^		n(%)	*OR*(95%*CI*)^c^		*ROR* ^#^
FCR							
High	1526(81.4)	1.00		1701(81.4)	1.00		1.00
Low	348(18.6)	1.58(1.38–1.82)^*^		389(18.6)	1.50(1.32–1.71)^*^		1.12(0.94–1.34)
MCR							
High	1332(71.1)	1.00		1456(69.7)	1.00		
Low	542(28.9)	1.39(1.22–1.58)^*^		634(30.3)	1.51(1.35–1.70)^*^		0.90(0.77–1.06)
PMPU							
No	1156(61.7)	1.00		1337(64.0)	1.00		
Yes	718(38.3)	2.15(1.91–2.43)^*^		753(36.0)	1.67(1.50–1.87)^*^		1.24(1.06–1.44)^*^

Note: FCR = father-child relationship; MCR = mother-child relationship; PMPU = problematic mobile phone use; NSSI = non-suicidal self-injury. ^c^ Adjusted for sex, boarding school, single child, grade, residency, family economic, parents’ education level, number of friends, FCR, MCR and PMPU; ^#^ Calculated by adjusted odds ratio. ^*^P < 0.001.

In addition, following reviewer suggestions, we also divided the sample into 4 groups, as high FCR + high MCR, low FCR + low MCR, high FCR + low MCR, and low FCR + high MCR. The results showed that in adjusted model, participants with low FCR + low MCR group were most associated with NSSI (OR = 2.96, 95% CI: 2.63–3.34) in the total sample, followed by the low FCR + high MCR group (OR = 2.32, 95% CI: 1.90–2.84), high FCR + low MCR (OR = 1.63, 95% CI: 1.47–1.82). Similar results were found in males and females, see Table S2 for details.

### Combined effect of PCR and PMPU on NSSI, and sex difference

PCR and PMPU were highly correlated in the present study (Table S3), therefore, we further analyzed the combined effect between PCR and PMPU on NSSI. The combined variables were divided into 4 groups: high FCR/MCR + no PMPU (reference group), low FCR/MCR + no PMPU, high FCR/MCR + yes PMPU, and low FCR/MCR + yes PMPU.

**Fig. 1:**
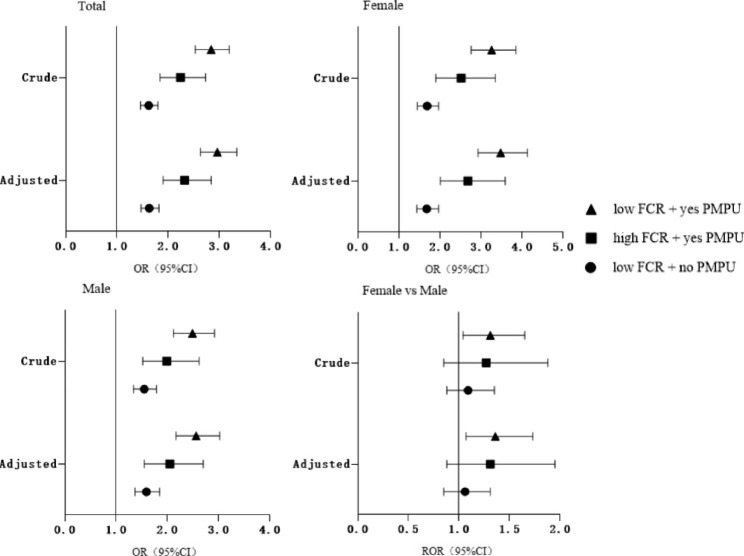
OR(95%CI) associated with the interaction of FCR and PMPU on NSSI in female and male, and the sex ratio. FCR = father-child relationship; PMPU = problematic mobile phone use; NSSI = non-suicidal self-injury. Adjusted for sex, boarding school, single child, grade, residency, family economic, parents’ education level, number of friends and and mother-child relationship

Figure 1 showed the results of 4 groups of FCR quality and PMPU in all participants, males, females and the sex comparison. In adjusted model, participants with low FCR + yes PMPU group were most associated with NSSI (OR = 2.96, 95% CI: 2.63–3.34) in the total sample, followed by the high FCR + yes PMPU group (OR = 2.32, 95% CI: 1.90–2.84), low FCR + no PMPU (OR = 1.63, 95% CI: 1.47–1.82). Similar results were found in males and females. Moreover, in low FCR + yes PMPU group, females had a higher risk of NSSI than males (ROR = 1.36, 95% CI: 1.07–1.73).

**Fig. 2:**
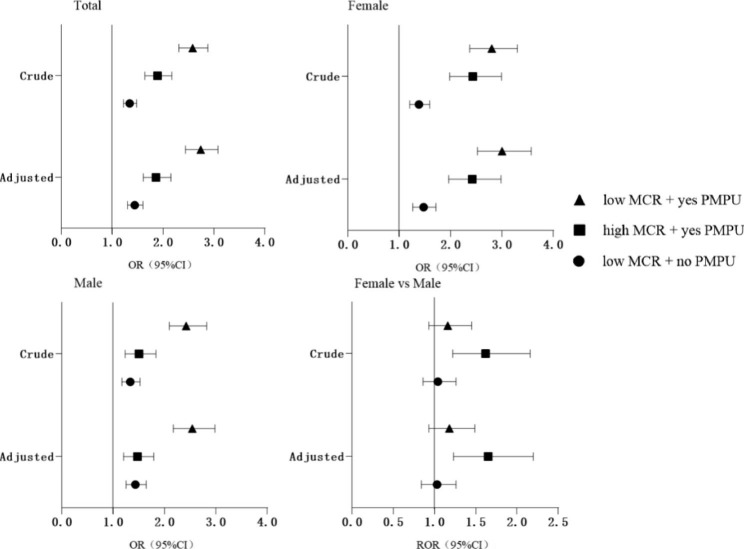
OR(95%CI) associated with the interaction of MCR and PMPU on NSSI in female and male, and the sex ratio. MCR = mother-child relationship; PMPU = problematic mobile phone use; NSSI = non-suicidal self-injury. Adjusted for sex, boarding school, single child, grade, residency, family economic, parents’ education level, number of friends and father-child relationship

Figure 2 showed the results of 4 groups of MCR quality and PMPU in all participants, males, females and the sex comparison. In adjusted model, participants with low MCR + yes PMPU were most associated with NSSI (OR = 2.74, 95% CI: 2.44–3.08) in the total sample, followed by the high MCR + yes PMPU (OR = 1.86, 95% CI: 1.61–2.15), low MCR + no PMPU (OR = 1.44, 95% CI: 1.30–1.60). Similar results were found in males and females. Moreover, in high MCR + yes PMPU group, females had a higher risk of NSSI than males (ROR = 1.65, 95% CI: 1.23–2.20).

### Interaction effect of PCR and PMPU on NSSI, and sex difference

Then we further analyze the additive interaction. After adjusting for confounding factors, the results in Table 4 showed that there was a positive additive interaction (synergistic effect) between MCR quality and PMPU on NSSI [*RERI* = 0.444 (0.115–0.772), *AP* = 0.162 (0.049–0.275), S*I* = 1.342 (1.062–1.694)]. However, there were no additive interaction effects between FCR quality and PMPU on NSSI, although they highly correlated to NSSI in the present study (Table S4). As shown in Table 5, among males, low MCR quality and PMPU additive interaction was associated with NSSI in adolescents [*RERI* = 0.646(0.219–1.074), *AP* = 0.254 (0.103–0.406), S*I* = 1.722 (1.136–2.609)]. However, among females, there were no additive interaction effects between MCR quality and PMPU on NSSI (Table S5).

**Table 4:** Additive interaction between mother-child relationship and PMPU with NSSI.

Model	Group		n(%)	*β*	*OR*(95%*CI*)	*RERI*	*AP*	*SI*
	MCR	PMPU						
Crude ^a^	High	No	3785(26.1)		1.00			
	Low	No	6775(46.7)	0.294	1.342(1.219–1.477)^*^			
	High	Yes	1258(8.7)	0.634	1.886(1.636–2.174)^*^			
	Low	Yes	2682(18.5)	0.948	2.579(2.309–2.881)^*^	0.354(0.090–0.618)	0.137(0.036–0.238)	1.288(1.042–1.593)
Adjusted ^b^	High	No	3785(26.1)		1.00			
	Low	No	6775(46.7)	0.366	1.442(1.303–1.596)^*^			
	High	Yes	1258(8.7)	0.619	1.857(1.607–2.146)^*^			
	Low	Yes	2682(18.5)	1.009	2.742(2.440–3.081)^*^	0.444(0.115–0.772)	0.162(0.049–0.275)	1.342(1.062–1.694)

Note: ^*^P < 0.001; MCR = mother-child relationship; PMPU = problematic mobile phone use; NSSI = non-suicidal self-injury. ^a^ Unadjusted model; ^b^ Adjusted for sex, boarding school, single child, grade, residency, family economic, parents’ education level, number of friends and father-child relationship.

**Table 5:** Additive interaction between mother-son relationship and PMPU with NSSI.

Model	Group		n(%)	*β*	*OR*(95%*CI*)	*RERI*	*AP*	*SI*
	MCR	PMPU						
Crude ^a^	High	No	2019(27.5)		1.00			
	Low	No	3291(44.8)	0.287	1.333(1.170–1.518)^*^			
	High	Yes	650(8.8)	0.403	1.496(1.226–1.825)^*^			
	Low	Yes	1387(18.9)	0.885	2.423(2.085–2.817)^*^	0.594(0.225–0.964)	0.245(0.104–0.386)	1.717(1.144–2.577)
Adjusted ^b^	High	No	2019(27.5)		1.00			
	Low	No	3291(44.8)	0.357	1.429(1.247–1.639)^*^			
	High	Yes	650(8.8)	0.383	1.466(1.199–1.793)^*^			
	Low	Yes	1387(18.9)	0.933	2.542(2.170–2.978)^*^	0.646(0.219–1.074)	0.254(0.103–0.406)	1.722(1.136–2.609)

Note: ^*^P < 0.001; MCR = mother-child relationship; PMPU = problematic mobile phone use; NSSI = non-suicidal self-injury. ^a^ Unadjusted model; ^b^ Adjusted for sex, boarding school, single child, grade, residency, family economic, parents’ education level, number of friends and father-child relationship.

## Discussion

### Main findings

Recent studies have revealed a high prevalence of NSSI among students in China. A Meta analysis included 420 studies with 160,348 Chinese middle school and high school students, and the results showed that the pooled rate of NSSI in a 6–24-month duration was 23.3% (95% CI: 20.5–26.1%) [40]. Moreover, Tang et al. indicated that in a sample of 15,623 Chinese students, 28.6% had NSSI in the past 12 months [41]. The results of this study are similar to that of the above study. But much higher than studies in the United States [3]. It may be related to the survey time and population, sociocultural background, questionnaire and evaluation method. In Chinese collectivisti culture, the interpersonal connectedness is highly valued and the interpersonal model may be a particularly relevant framework for understanding NSSI development among Chinese adolescents [42]. At the same time, we also found that middle school students had a higher incidence of NSSI than high school students. In other words, NSSI is more likely to occur in early adolescence, which corroborates with the results of other studies [20, 43]. This may be explained by the fact that adolescence is a critical period of development characterized by increased risky behaviors including NSSI. The possible mechanism is heightened reward-related brain activation and relatively limited recruitment of prefrontal regions. A previous study pointed out that youth with NSSI behaviors showed higher activation in response to bilateral monetary reward, and the heightened neural sensitivity to reward was related to NSSI ideas in early adolescence [44]. Hence, the prevention and control of NSSI behaviors should be implemented as early as possible.

Our study found that lower paternal relationship and maternal relationship quality were associated with NSSI, even after adjusting for sociodemographic risk factors. This conforms with the standpoints of Linehan’s theoretical model for the development of borderline personality disorder, self-harming and suicidal behaviors [45], which held that an invalidating environment during childhood is a substantial risk factor in the etiology of NSSI, and the overall quality of relationships with parents in childhood, disruption of the PCR, and experiences of separation and loss may lead to later self-harm behaviors [46]. Previous studies also supported that high-quality PCR is a protective factor, and the parent-child relational risk can have a potential impact on the adolescents’ self-injury behavior [47, 48]. This evidence suggests that higher PCR quality is a fundamental factor in preventing self-injury behavior in adolescents. Furthermore, our investigation reports that adolescents with PMPU have a higher risk of NSSI, which is consistent with previous research [20, 49]. A study of community sample reveals that smartphone addiction could positively predict NSSI, particularly in pre-adolescence as compared to adolescents, which was related to low self-control and emotion dysregulation [49]. However, differently, contrary to this study, was that there was no sex difference between smartphone use and self-injury [49, 50]. The results of this study further expand the current understanding of the relationship between PMPU and NSSI among adolescents and highlight the need to focus on females.

Additionally, we further analyzed the combined effect between PCR and PMPU on NSSI, among which, the low PCR + yes PMPU group had the highest association with NSSI. Overall, the association was stronger among females than males. A prospective-longitudinal community-representative study reported that cumulative exposure to more adverse life events were associated with an increased risk of self-injury throughout adolescence, females were more likely than males to use self-injury when faced with stressful events in school and intimate relationships [51]. In fact, studies have found that adolescent female tend to be more sensitive to relationship stressors than male [52]. This is consistent with Nock’s theory that self-injury is usually a response to stressful experiences [53]. Adverse life events may cause excessive stress in adolescent female, which in turn triggers self-harm, a maladaptive strategy for relieving distress. Among females, self-injury can be a coping mechanism that fits sex stereotypes and is easily acquired. Among males, most of them may choose more male-typical maladaptive coping behaviors such as substance use, NSSI may not conform to typical male behavior because it is less common in male peer groups [51, 53].

Finally, we explored the additive interaction between PCR and PMPU on NSSI, respectively. The study found that the interaction between lower MCR and PMPU was associated with NSSI. This finding was in accordance with the traditional Chinese family culture background; wherein the fathers are usually more career-oriented because of the economic pressure and social factors, while the mothers are more involved in parenting and accompanying their children [54]. Lan et al. [55] underscored the centrality of mothers in the child-rearing process within Chinese families. Studies have found that parents play different roles in parent-child interactions [17]. Predominantly, fathers spend relatively less time interacting with their children, while mothers devote more time and energy to participate in caring for their child and family. As the children grow up, mothers tend to be more responsive to them and more altruistic in parenting [56]. In addition, Sbarra et al. [57] introduced and outlined the case of an evolutionary mismatch between smartphones and social behaviors that help form and maintain close social relationships [58]. Thus, although our lives have become more convenient because of mobile phones, they have contributed to a series of adverse consequences such as causing people to fall into the trap of virtual/online world, which further disrupts their real-life interpersonal interactions. Meanwhile, researchers found that mothers also showed high levels of control and rejection, in terms of enforcing discipline with their children [58]. Children also believe that parent-child conflicts are more with their mothers than with their fathers [54]. Since mothers take on a more prominent parenting role in Chinese families, negative maternal relationship may have a greater impact on Chinese adolescents. Therefore, these findings indicated that the role of lower maternal relationship quality and PMPU should be pay more attention in Chinese adolescents. Understanding these interactions will help make progress in strengthening and facilitating the development of prevention strategies for NSSI.

### Strengths and limitations

This was a large-scale, school-based study to examine the independent, combined and additive interaction effects of PCR quality and PMPU on NSSI among adolescents in China, and sex differences in these associations. The sample was well representative, balancing China’s urban and rural areas, grade distribution, and so on. In particular, these findings shed light on the differences in the impacts of fathering and mothering on the lives of adolescents in different sexes, which were neglected in the scientific literature.

However, there are several limitations that must be taken into consideration when interpreting the results. First, this study was a cross-sectional survey; hence, the causal relationship between lower PCR quality and PMPU dependence and NSSI is not clear, and further cohort studies are necessary to understand this. Second, the measurement of such behaviors is fraught with methodological challenges. For example, many of the variables used in this study are retrospective and self-report measures, which may be affected by recall bias and common variance problems. Currently, for studies involving adolescent sample, there is a tendency to rely on a single informant report, which leads to greater subjective. Importantly, some recent findings have identified inconsistencies in data across multiple information providers.

[59, 60]. Therefore, it is recommended that future researches attempt to take the PCR quality report by both individual and their parents. Third, the topics of PCR quality and NSSI behavior were relatively sensitive, which may affect the authenticity of the participants’ responses to questions. Therefore, our results may represent a more conservative estimate than is actually the case. Finally, the etiology of NSSI in adolescents involves many aspects. Current theories suggest that the emotional instability displayed by self-harming adolescents is caused by the complex interaction between individual biological vulnerability and environmental risk [61, 62]. For example, insufficient serotonin function coupled with certain family patterns, among other factors, may have an impact on an individual’s self-harming tendencies. We could not possibly consider all variables at play in our analyses, and perhaps unmeasured factors could also explain variables in NSSI outcomes. Therefore, in-depth exploration is required in the future.

## Conclusions

In conclusion, this study showed that lower PCR quality and PMPU were independently and combinedly associated with NSSI. In some groups, there were sex differences. Moreover, the addictive interaction effect between lower maternal relationship quality and PMPU was associated with NSSI, whereas, there was no interaction effect between lower paternal relationship quality and PMPU on NSSI. Therefore, the results further emphasize that studying the influencing factors of PCR quality and the emerging PMPU simultaneously contributes to a comprehensive understanding of NSSI and may help with the prevention and control of NSSI in adolescents.

## Electronic supplementary material

Below is the link to the electronic supplementary material.

**Supplementary file 1**: **Table S1**: Distribution of the non-suicidal self-injury methods. **Table S2**: Number, percent and odds ratio of NSSI by different groups of father-child relationship and mother-child relationship in the total sample. **Table S3**: The prevalence of PMPU by level of parent-child relationship, n(%). **Table S4**: Additive interaction between father-child relationship and PMPU with NSSI. **Table S5**: Additive interaction between mother-daughter relationship and PMPU with NSSI.

## Data Availability

The datasets analyzed in this study are not yet publicly available. Requests to access the datasets should be directed to 2,004,500,039@ahmu.edu.cn.
