# Supplementary material for: Association of parent-child relationship quality and problematic mobile phone use with non-suicidal self-injury among adolescents
Source: BMC Psychiatry. 2023 May 1;23:304. doi: 10.1186/s12888-023-04786-w (PMC10152594; doi:10.1186/s12888-023-04786-w)
Supplement: Supplementary file 1 — Table S1: Distribution of the non-suicidal self-injury methods. Table S2: Number, percent and odds ratio of NSSI by different groups of father-child relationship and mother-child relationship in the total sample. Table S3: The prevalence of PMPU by level of parent-child relationship, n(%). Table S4: Additive interaction between father-child relationship and PMPU with NSSI. Table S5: Additive interaction between mother-daughter relationship and PMPU with NSSI. [file 12888_2023_4786_MOESM1_ESM.docx]

**Supplemental files**

**Table S1 Distribution of the non-suicidal self-injury methods.**

| Method | n | % |
| --- | --- | --- |
| 1. pinched yourself | 2178 | 15.0 |
| 1. scratched yourself | 1421 | 9.8 |
| 1. banged your head | 1433 | 9.9 |
| 1. fist against something | 3018 | 20.8 |
| 1. hit yourself | 1159 | 8.0 |
| 1. prick or stab yourself | 781 | 5.4 |
| 1. cut yourself | 738 | 5.1 |
| 1. bitten yourself | 1123 | 7.7 |
| 1. pulled your own hair | 1453 | 10.0 |
| 1. burned yourself | 207 | 1.4 |
| 1. rub skin to bleed or bruise | 560 | 3.9 |
| 1. engrave words or symbols on the skin | 1358 | 9.4 |

**TableS2 Number, percent and odds ratio of NSSI by different groups of father-child relationship and mother-child relationship in the total sample.**

| Group | | n(%) | Model 1 |  | Model 2 |  | Model 3 |
| --- | --- | --- | --- | --- | --- | --- | --- |
| FCR | MCR |  | *OR*(95%*CI*)^a^ |  | *OR*(95%*CI*)^b^ |  | *OR*(95%*CI*)^c^ |
| High | High | 1002(6.9) | 1.00 |  | 1.00 |  | 1.00 |
| Low | Low | 6805(46.9) | 2.924(2.428-3.520)^*^ |  | 2.840(2.351-3.431)^*^ |  | 2.548(2.107-3.083)^*^ |
| High | Low | 2652(18.3) | 1.789(1.464-2.186)^*^ |  | 1.770(1.446-2.166)^*^ |  | 1.745(1.424-2.137)^*^ |
| Low | High | 4041(27.9) | 2.123(1.752-2.572)^*^ |  | 1.985(1.635-2.409)^*^ |  | 1.825(1.502-2.218)^*^ |
| Note: FCR=father-child relationship; MCR=mother-child relationship; NSSI=non-suicidal self-injury. ^a^ Unadjusted model; ^b^ Adjusted for sex, boarding school, single child, grade, residency, family economic, parents’ education level, number of friends. ^c^ Adjusted for sex, boarding school, single child, grade, residency, family economic, parents’ education level, number of friends, and problematic mobile phone use. ^*^ *P*<0.001. | | | | | | | |

**Table S3 The prevalence of PMPU by level of parent-child relationship, n(%)**

| Parent-child relationship | PMPU | | *P*-Value^*^ |
| --- | --- | --- | --- |
|  | no | yes |  |
| FCR |  |  |  |
| High | 3076(84.2) | 578(15.8) | <0.001 |
| Low | 7484(69.0) | 3362(31.0) |  |
| MCR |  |  |  |
| High | 3785(75.1) | 1258(24.9) | <0.001 |
| Low | 6775(71.6) | 2682(28.4) |  |
| Note: FCR=father-child relationship; MCR=mother-child relationship; PMPU=problematic mobile phone use. ^*^ trend P value | | | |

**Table S4 Additive interaction between father-child relationship and PMPU with NSSI.**

| Model | Group | | n(%) | *β* | *OR*(95%*CI*) | *RERI* | *AP* | *SI* |
| --- | --- | --- | --- | --- | --- | --- | --- | --- |
|  | FCR | PMPU |  |  |  |  |  |  |
| Crude ^a^ | High | No | 3076(21.2) |  | 1.00 |  |  |  |
|  | Low | No | 7484(51.6) | 0.482 | 1.620(1.457-1.801)^*^ |  |  |  |
|  | High | Yes | 578(4.0) | 0.807 | 2.241(1.841-2.729)^*^ |  |  |  |
|  | Low | Yes | 3362(23.2) | 1.045 | 2.843(2.533-3.192)^*^ | -0.017(-0.468-0.434) | -0.006(-0.165-0.153) | 0.991(0.777-1.263) |
| Adjusted ^b^ | High | No | 3076(21.2) |  | 1.00 |  |  |  |
|  | Low | No | 7484(51.6) | 0.490 | 1.633(1.466-1.820)^*^ |  |  |  |
|  | High | Yes | 578(4.0) | 0.844 | 2.324(1.904-2.839)^*^ |  |  |  |
|  | Low | Yes | 3362(23.2) | 1.086 | 2.963(2.630-3.338)^*^ | 0.004(-0.497-0.506) | 0.001(-0.168-0.171) | 1.002(0.776-1.295) |
| Note: ^*^*P*<0.001; FCR=father-child relationship; PMPU=problematic mobile phone use; NSSI=non-suicidal self-injury. ^a^ Unadjusted model; ^b^ Adjusted for gender, boarding school, single child, grade, residency, family economic, parents’ education level, number of friends and mother-child relationship. | | | | | | | | |

**Table S5 Additive interaction between mother-daughter relationship and PMPU with NSSI.**

| Model | Group | | n(%) | *β* | *OR*(95%*CI*) | *RERI* | *AP* | *SI* |
| --- | --- | --- | --- | --- | --- | --- | --- | --- |
|  | MCR | PMPU |  |  |  |  |  |  |
| Crude ^a^ | High | No | 1766(24.7) |  | 1.00 |  |  |  |
|  | Low | No | 3484(48.7) | 0.323 | 1.381(1.197-1.594)^*^ |  |  |  |
|  | High | Yes | 608(8.5) | 0.890 | 2.434(1.983-2.988)^*^ |  |  |  |
|  | Low | Yes | 1295(18.1) | 1.028 | 2.796(2.373-3.295)^*^ | -0.021(-0.543-0.501) | -0.007(-0.195-0.180) | 0.988(0.741-1.319) |
| Adjusted ^b^ | High | No | 1766(24.7) |  | 1.00 |  |  |  |
|  | Low | No | 3484(48.7) | 0.383 | 1.467(1.260-1.709)^*^ |  |  |  |
|  | High | Yes | 608(8.5) | 0.883 | 2.417(1.958-2.984)^*^ |  |  |  |
|  | Low | Yes | 1295(18.1) | 1.099 | 3.002(2.524-3.572)^*^ | 0.116(-0.472-0.705) | 0.039(-0.155-0.233) | 1.062(0.781-1.444) |
| Note: ^*^*P*<0.001; MCR=mother-child relationship; PMPU=problematic mobile phone use; NSSI=non-suicidal self-injury. ^a^ Unadjusted model; ^b^ Adjusted for gender, boarding school, single child, grade, residency, family economic, parents’ education level, number of friends and father-child relationship. | | | | | | | | |
